# The Roles of Junctional Adhesion Molecules (JAMs) in Cell Migration

**DOI:** 10.3389/fcell.2022.843671

**Published:** 2022-03-09

**Authors:** Junqi Wang, Han Liu

**Affiliations:** ^1^ Beijing Proteome Research Center, Beijing Institute of Lifeomics, Beijing, China; ^2^ Department of Pharmacy, People’s Hospital of Longhua, Shenzhen, China

**Keywords:** junctional adhesion molecules, cell migration, germ cell, epithelial cells, endothelial cells, leukocytes, cancer cells

## Abstract

The review briefly summarizes the role of the family of adhesion molecules, JAMs (junctional adhesion molecules), in various cell migration, covering germ cells, epithelial cells, endothelial cells, several leukocytes, and different cancer cells. These functions affect multiple diseases, including reproductive diseases, inflammation-related diseases, cardiovascular diseases, and cancers. JAMs bind to both similar and dissimilar proteins and take both similar and dissimilar effects on different cells. Concluding relevant results provides a reference to further research.

## Introduction

Cell migration plays a pivotal role in tissue organization during development, and several diseases develop due to its dysregulation. The migration properties are partly dictated by cell adhesion and its endocytic regulation, and scaffold cell-dependent migration could be represented among various types of migration (Kawauchi 2012). It is critical to maintain junctional integrity during cell migration and cell extrusion through classic cell–cell junctions member protein such as ZO-1 (Garcia et al., 2018). Transmembrane and associated cytoplasmic proteins in tight junctions show dynamic behavior, including migration within the junction and exchange in and out of the junctions (Chalmers and Whitley 2012). In addition, internalized tight junctions (TJs) proteins are recycled to the plasma membrane or sorted to late endosomes and degradation (Stamatovic et al., 2017).

Junctional adhesion molecule (JAM) is a member of the immunoglobulin superfamily localized at the tight junction of polarized cells and on the cell surface of leukocytes (Ebnet et al., 2004). Several members of the family mediate cell polarity, endothelium permeability, and leukocytes migration through a multitude of homophilic and heterophilic interactions with intrafamily and extrafamily partners (Garrido-Urbani et al., 2014). Several members of the JAM family interact with PDZ domain-containing scaffolding proteins such as ZO-1, claudin, and afadin, regulating cell–cell contact maturation and the generation of junctional complexes such as TJs and adherens junctions (AJs) (Keiper et al., 2005a; Ebnet 2017; Hartmann et al., 2020). In the early years, there existed some inconsistencies in the JAM nomenclature. In this review, we quote the original description of these articles and specific information referring to the review of Mandell and Parkos (Mandell and Parkos 2005).

### Germ Cell Motility, Polarization, and Maturation

Germ cells (GCs) migrate spatially distinct locations for proper development with various patterns; nevertheless, the reason and cellular mechanisms that facilitate germ cells motility and guide migration *in vivo* remain unclear (Kanamori et al., 2019; Grimaldi and Raz 2020). The adhesive interaction between germ and Sertoli cells (SCs) regulates spermatogenesis (Cartier-Michaud et al., 2017).

Among all members and family-related molecules, JAM-C could be the most striking protein modulating germ cell activities. JAM-C localizes to germ/Sertoli cell contacts and participates in acrosome formation and germ cell polarity, specifically round spermatids. Intriguingly, JAM-C is restricted to the apical ectoplasmic specialization (ES) but not at the blood–testis barrier (BTB) in mouse testes. Par6, Cdc42, PKCl, and PATJ are identified as the downstream of engaged JAM-C protein, mediating spermatid polarization in mice and possibly in humans together (Gliki et al., 2004). Furthermore, Par6 formed a stable complex with Pals1 and JAM-C in normal testes, and the tight association of the Par6/Pals1 complex with Src kinase rendered a loss of association of the Par6/Pals1 complex with JAM-C, thereby destabilizing apical ES to facilitate spermatid loss (Wong et al., 2008). Additionally, RA175 formed a ternary complex with JAM-C *via* interaction with Par-3, which may take effect when the specialized adhesion structures of elongating spermatid form (Fujita et al., 2007). Another demonstrated partner of JAM-C is CAR, and they both are components of apical ES involved in spermatid orientation, facilitating cell movement and orientation in the seminiferous epithelium (Mirza et al., 2006; Yan et al., 2007). Unfortunately, another research instead implied that JAM-C controlled germ cell differentiation without reference to CAR and the interaction between CAR and JAML, meanwhile, appears not to confer transepithelial migration of cells in the BTB (Sultana et al., 2014). *Jam-C*-deficient males are infertile and fail to produce mature sperm cells with about 50% smaller testes and lacks differentiated elongated spermatids (Gliki et al., 2004). Similarly, Spo11(Cre) mice crossed with floxed JAM-C mice to produce conditional knockouts and showed a strong reduction of JAM-C protein levels in the testis and a spermiogenetic arrest (Pellegrini et al., 2011).

JAM-A is present in the prostate and seminal vesicles and all three regions of the epididymis. JAM-A is located at both Sertoli and a subset of basal GCs, specifically in inter-Sertoli cell junctions and the tails of elongated spermatids within the epididymis of rodents, secreted in epididymosomes in the luminal fluid, and delivered to sperm *in vitro* (Shao et al., 2008; Wu et al., 2017). When the Sertoli cell tight junction was perturbed *in vitro*, BTB-associated proteins JAM-A disappear from the cell–cell interface (Xia et al., 2007). At the time of germ cell migration across the BTB during spermatogenesis, the TJ- and AJ-integral membrane proteins (including JAM-1) can be disengaged to facilitate AJ restructuring, accommodating germ cell migration while maintaining the BTB integrity (Yan and Cheng 2005). JAM-A expressed in premeiotic GCs facilitates GC migration through the BTB and then disappears in most GCs resident in the adluminal compartment, and CAR has mechanisms similar to localization and involvement in GC migration (Wang and Cheng 2007; Wang et al., 2007; Tarulli et al., 2008). JAM-A reactivity declined in Sertoli cells from tubules with testicular carcinoma *in situ* (CIS) and emerged to be strong in seminoma (Tarulli et al., 2013). sGCb1 plays an important role in the restructuring of the adherent junction in the testis, which seems associated with JAM-A (Sarkar et al., 2006). The presence of TGF-β3 and TNFα enhanced the kinetics of endocytosis of JAM-A from the Sertoli cell surface, and TGF-β3 disrupted JAM-A-based TJ fibrils (Xia et al., 2009). More association between P-glycoprotein (P-gp, MDR1) and JAM-A possibly enhanced BTB function (Su et al., 2009). In sperm, Ca^2+^ homeostasis is sustained by the relative ratios of CASK–PMCA4b and CASK–JAM-A interactions, as JAM-A positively regulates PMCA4b by sequestering CASK under conditions of elevated Ca^2+^ (Aravindan et al., 2012). Multiple stimuli have been shown to affect JAM-A function. Cyproterone acetate (CPA) could somehow reduce the kinetics of internalization of JAM-A, whose maintenance impliedly requires a very low level of endogenous testosterone (Yan et al., 2008). C-type natriuretic peptide (CNP) produced by Sertoli and germ cells into the BTB microenvironment accelerates endocytosis of JAM-A and opens the BTB transiently to encourage preleptotene spermatocyte migration (Xia et al., 2007). Excess iodine causes loss of spermatogenesis by disrupting the blood–testis barrier and cytoskeleton, with reduced expression of JAM-A in blood–testis barrier proteins (Chakraborty et al., 2020). The commercial polychlorinated biphenyls mixture, Aroclor1254 treatment, could induce increments in JAM-A endocytosis and occludin ubiquitination in primary cultured SCs and rats (Jia et al., 2017). In addition, oxidative damage participates in heat stress-induced downregulation of tight junction proteins in Sertoli cells by inhibiting the CaMKKβ–AMPK axis in boars, which was reversed by N-acetyl-l-cysteine (NAC) (Yang et al., 2020). IL-6 treatment delayed the kinetics of JAM-A, leading to accumulation in Sertoli cells (Zhang et al., 2014). Presently, JAM-A has been regarded as typical BTB-associated integral membrane proteins and several other related proteins mainly including tight junction, ectoplasmic specialization, and adherens junction proteins.

In the mammalian testis, JAM-B occurs in the blood–testis barrier between Sertoli cells and the apical ectoplasmic specializations between Sertoli and germ cells, promoting the transit of developing germ cells across the blood–testis barrier and the timely release of mature spermatids at stage VIII. In MSC-1 cells, the binding of various transcription factors to various cis-acting elements-binding motifs regulates the constitutive expression of JAM-B (Wang and Lui 2009). IL-1alpha promotes JAM-B expression by facilitating the binding of Elk-1 to TG-interacting factor (TGIF) and proximal Sp1 (pSp1) + E2F motifs in a p38-dependent manner, leading to an additive effect on Sp1-and neuron-restrictive silencer factor (NRSF)-mediated JAM-B transactivation (Wang and Lui 2009). Rather, TGF-beta2 inhibits JAM-B transcription *via* the activation of mothers against decapentaplegic (Smad) proteins, and activated Smads compete with specificity proteins (Sp1 and Sp3) for the TGIF motif, causing JAM-B repression (Wang and Lui 2009). Transforming growth factor-β3 regulates cell junction restructuring *via* Smad-dependent protein degradation of JAM-B (Zhang and Lui 2015). JAM-A and JAM-B are localized at the BTB, and JAM-C on spermatids interact with JAM-B on the Sertoli cell to foster the morphological polarization of round spermatids to elongated spermatids (Ebnet 2017). Graspin inhibited the PDZ-mediated interactions of GRASP55 with JAMs, resulting in hampered polarized localization of JAM-C in spermatids, the premature release of spermatids, and the affected Golgi morphology of meiotic spermatocytes (Cartier-Michaud et al., 2017). Another member JAM4 protein could function as a cell adhesion molecule rather than a tight-junction protein in the testis during BTB formation; generally, JAM might participate in homophilic cell adhesion between spermatogonia–spermatogonia, spermatogonia–Sertoli cells, and Sertoli cells–Sertoli cells (Nagamatsu et al., 2006).

Germ cells have impressive and special migration characteristics. In adult rat testes, the blood–testis barrier, TJs between Sertoli cells (Byers et al., 1991), in the seminiferous epithelium must “open” (or “disassemble”) to accommodate the migration of preleptotene spermatocytes from the basal to the adluminal compartment that occurs at stage VIII of the epithelial cycle (Xia et al., 2007). To recap, transepithelial migration of male germ cells across the BTB regulates sperm motility and spermatid differentiation, and JAMs have an exact function to regulate it. The roles of JAMs in germ cell motility, polarization, and maturation have been concluded in Table 1.

**TABLE 1:** JAMs in germ cell motility, polarization, and maturation.

Protein	Location	Partner	Final effect
JAM-A	Sertoli cell and a subset of basal GCs	P-glycoprotein	Maintains the BTB integrity
		Su et al. (2009)	
		CASK	
		Aravindan et al. (2012)	
JAM-B	BTB between Sertoli and germ cells	JAM-C on spermatids	Promotes germ cells to transit across the BTB
		Ebnet (2017)	Wang and Lui (2009)
JAM-C	Germ/Sertoli cell	Par6/Pals	Mediates spermatid polarization and differentiation and produces mature sperm cells
		Wong et al. (2008)	
		Par-3	
		Fujita et al. (2007)	
		CAR	
		Sultana et al. (2014)	

### Epithelial and Epidermis Barrier

Keratinocytes, the predominant cell type of the epidermis, migrate to bring tissue reepithelialization and reinstate the epithelial barrier during wound healing (Garcia et al., 2018; Holt et al., 2021). JAM-A is expressed in epidermis two-folds higher than that in full-thickness skin predominantly located at the cell–cell interface in the epidermis (Wang et al., 2018). JAM-A knockdown promotes keratinocyte proliferation and migration to improve the skin healing process *in vivo*, regulated by the signaling of FAK-mediated Erk1/2 activation (Wang et al., 2018). Bioactive glass (BG) extracts a posttranscriptional regulation mechanism on the expression of JAM-A to assemble into TJs located along the edge of the cell membrane, involved in mediating the enhanced barrier function of the keratinocyte monolayers (Tang et al., 2021).

JAM-A cis-homodimers encourage the formation of a complex with afadin and PDZ-GEF2 to enhance cell migration by activating the small GTPase Rap1A, whose active levels are decreased by JAM-A depletion or overexpression of cis-dimerization mutants (Mandell et al., 2005; Severson et al., 2008; Severson and Parkos 2009a; Severson and Parkos 2009b; Severson et al., 2009; Luissint et al., 2014). Additionally, Rap1 activity activation requires N-glycosylation of JAM-A, reinforcing barrier function, as glycosylation of N185 is required for JAM-A-mediated reduction of cell migration (Scott et al., 2015). Interestingly, trans-null but not cis-null JAM-A mutant expression decreased Rap2 activity, which implies trans-dimerization of JAM-A as a barrier-inducing molecular switch (Monteiro et al., 2014). A functional complex comprising JAM-A, α3β1 integrin, and tetraspanins CD151 and CD9 regulated collective cell migration of polarized epithelial cells (Thölmann et al., 2022). JAM-A protein is found at the leading edge of repairing corneal epithelial wounds in wild-type mice, while corneal epithelial wound repair was qualitatively normal, but corneal epithelium cells are irregularly shaped in JAM-A null animals (Kang et al., 2007). JAM-A deletion worsened intestinal hyperpermeability and therefore increases intestinal epithelial migration (Meng et al., 2019). Similarly, in oral epithelial cells, ligation of CD24 induces a c-Src kinase-dependent decrease in paracellular permeability mediated by JAM-A and other tight junction proteins, which also affects migrating epithelium of the periodontitis lesion (Ye et al., 2011). The increase in JAM-A expression following Ykt6 knockdown drives prostate epithelial cell motility by stimulating Rap1 and Rac1 small GTPases, regulated by miR-145 (Naydenov et al., 2018).

Alternatively, JAM-C regulates epithelial cell migration at the level of *β1* integrin activity but not integrin expression (Mandicourt et al., 2007; Ebnet 2017). JAM-C distributed nonclassically in the apical membranes of Müller cells and retinal pigment epithelial (RPE) (Daniele et al., 2007; Economopoulou et al., 2009), and JAM-C knockdown inhibits human RPE cell migration but not proliferation and decreases the permeability of monolayer hRPE (Hou et al., 2012). JAM3 is expressed in multiciliated cells (MCCs) in the airway epithelium, and JAM3 lacking causes a delay in BB assembly/positioning during MCC differentiation (Mateos-Quiros et al., 2021).

Moreover, JAM-A was often observed for aberrant cytoplasmic expression in diseased gingival tissues and more expression within the leukocytes in disease-associated epithelia (Choi et al., 2014). γδ T cells present epithelial tissue bridge innate and adaptive immunity. More interestingly, the costimulation of JAML with its endogenous ligand CAR or by binding to the stimulatory antibody HL4E10 activates epithelial γδ T cells, leading to cellular proliferation, migration, and adhesion (Witherden et al., 2010; Verdino et al., 2011; Johnson et al., 2021).

Overall, JAMs regulate the migration process of multiple epithelial cells in different but connected ways, whose mechanisms remain to be explored. Conspicuously, JAMs play striking roles in cancer invasion and metastasis as discussed in the following chapter. The roles of JAMs in the epithelial and epidermis barrier have been concluded in Table 2.

**TABLE 2:** JAMs in the epithelial and epidermis barrier.

Protein	Cell type	Partner	Final effect
JAM-A	Epidermis, intestinal epithelial cells, oral epithelial cells, prostate epithelial cells, and MDCK cells	Afadin and PDZ	Inhibits cell migration and induces permeability
		Severson et al. (2009)	(Tang et al. (2021);
		α3β1 integrin	Meng et al. (2019);
		Thölmann et al. (2022)	Ye et al. (2011);
			Naydenov et al. (2018))
			Reduces collective cell motility
			(Thölmann et al. (2022))
JAM-C	Human RPE cell	*β1* integrin	Inhibits migration but not proliferation and decreases the permeability
		Hou et al. (2012)	(Hou et al. (2012))
JAML	Epithelial γδ T cell	CAR	Promotes proliferation, migration, and adhesion
		Witherden et al. (2010)	(Witherden et al. (2010);
			Verdino et al. (2011);
			Johnson et al. (2021))

### Endothelial and Endothelial Barrier

JAM-A regulates cell migration through changes in directional persistence under shear flow by cooperating with microtubule-stabilizing pathways in endothelial cells (ECs) (Huang et al., 2006; Severson and Parkos 2009a). Soluble JAM-A blocked cultured endothelial cells migration (Koenen et al., 2009). Signaling through JAM-A is necessary for alpha(v)beta(3)-dependent HUVEC migration, and this effect could be increased by engagement of the ligand-binding site of the integrin by Arg-Gly-Asp-Ser (RGDS) peptide and blocked by phosphoinositide 3-kinase and protein kinase C inhibitors (Naik and Naik 2006). The ternary JAM-A-CD9-αvβ3 integrin complex releases JAM-A upon bFGF stimulation to activate ERK and to regulate endothelial cell migration on vitronectin (Naik et al., 2003; Parise 2003; Cooke et al., 2006; Peddibhotla et al., 2013). Similar to its role in the epithelium, JAM-A dimerization works in close cytoplasmic apposition of complexes containing specific PDZ domain-containing scaffold proteins, which activates small G protein Rap1 to stabilize β1 integrin protein and promotes endothelial cell migration (Severson and Parkos 2009b). Furthermore, N-glycosylation of JAM-A contributes to Rap1 activity, and glycosylation of N185 is required for JAM-A-mediated reduction of cell migration (Scott et al., 2015). ZO-1 and JAM-A assemble into a cooperative unit and then induce the formation of actin/myosin II stress fibers and redistribution of vinculin and PAK2 from adherens junction to focal adhesions in primary EC, regulating endothelial cell migration and angiogenic potential (Tornavaca et al., 2015). Tight junction protein (JAM-A and ZO-1) expression suppressed by rosiglitazone, which is linked to promote endothelial cell migration and induced permeability resulting from rosiglitazone (Ku et al., 2017). miR-145-rich exosomes can inhibit the migration of HUVECs *via* targeting JAM-A (Yang et al., 2021). The inhibitory effects on cell migration of human retinal capillary endothelial cells (HRCECs) induced by high concentrations of glucose were reversed once the expression of secreted protein acidic and rich in cysteine (SPARC) was inhibited, possibly associated with increased expression of JAM1 (Fu et al., 2019). Incidentally, JAM-A is a prerequisite for inflamed SMCs migration (Azari et al., 2010).

Consistently, soluble matrix-bound forms of JAM-C could be instrumental in guiding migration and adhesion of hematopoietic cells and vascular endothelial cells to the limbal niche, but further studies are warranted to investigate the precise role of JAMs and other IgCAMs in the human limbus (Polisetti et al., 2016). sJAM-C stimulates human microvascular endothelial cell (HMVEC) migration *in vitro*, dependent on Src, p38, and PI3K (Rabquer et al., 2010). The ubiquitylation of JAM-C by the E3 ligase Casitas B-lineage lymphoma (CBL) and dynamic JAM-C trafficking and degradation are necessary for junctional remodeling during cell migration (Kostelnik et al., 2019). Over-expressed or hypoxia-induced miR-212/132 led to a downregulation of JAM-C in human brain microvascular endothelial cells (BMECs) and resulted in slower migration of BMECs (Burek et al., 2019). JAM-C, expressed by the tumor endothelium, is obligated to transvascular migration of embryonic-endothelial progenitor cells (e-EPCs) (Czabanka et al., 2020).

To conclude, various types of endothelial cells migrate with the regulation of JAMs related to diverse diseases. JAMs mediate a variety of immune cells. Transendothelial migration is another predominant function, and this review will discuss it in the next chapter.

## Immune Cells

JAMs governing various types of immune cell migration has been a project under the limelight, especially their function in transendothelial migration (TEM) and transepithelial migration (TEpM). The first and second immunoglobulin domains of JAM-A and the I domain of leukocyte function-associated antigen-1 (LFA-1) support the interaction of JAM-A snd LFA-1, which destabilizes the JAM-A homophilic interaction to promote LFA-1-dependent transendothelial migration of T cells and neutrophils (Ostermann et al., 2002; Fraemohs et al., 2004; Wojcikiewicz et al., 2009). N-glycosylation of JAM-A turned out to regulate leukocyte LFA-1 binding (Scott et al., 2015). Furthermore, phosphorylation of JAM-A at Ser-284 activated RhoA to facilitate leukocyte TEM through interactions with the integrin LFA-1, dependent on PI3K-mediated activation of GEF-H1 and p115 RhoGEF (Scott et al., 2016). JAM-A antagonist peptide (JAM-Ap) blocked the interaction of JAM-A with LFA on neutrophils and monocytes/macrophages and attenuated brain ischemia/reperfusion (I/R)-induced neutrophil and monocyte infiltration into the brain parenchyma (Sladojevic et al., 2014). Meanwhile, pro-inflammatory cytokines such as TNF-alpha and IFN-gamma induced JAM redistribution and might further promote TEM of leukocytes (Ozaki et al., 1999). Apart from the typical partnership with LFA-1, JAMs instruct TEM through other mechanisms as follows:

### Stem and Progenitor Cells

Wu et al. found that the JAM-A overexpression MSCs (JAM-A(ov) MSCs) migrated into the hair follicle (HF) sheath, and JAM-A promoted MSC proliferation and migration by activating T-cell lymphoma invasion and metastasis 1 (Tiam1) (Wu et al., 2014; Wu et al., 2015). Moreover, a JAM-C-blocking monoclonal antibody induces HSPC mobilization in a JAM-B dependent manner (Arcangeli et al., 2014).

### Neutrophil (PMN)

JAM-A mediated neutrophil migration through the endothelium, which is dependent on IL-1β stimulus and only required endothelial-cell JAM-A and not leukocyte JAM-A (Woodfin et al., 2007; Cera et al., 2009). Furthermore, JAM-A mediates TNF-alpha-induced neutrophil transmigration by activating leukocytes and endothelial cells *in vivo* (Woodfin et al., 2009). JAM-A exclusively secreted from cardiac progenitor cells (CPCs) inhibited the transmigration across inflamed endothelium into the myocardium and affected complement factor 5 (C5aR)-dependent function, reducing oxidative stress and inflammatory response after infarction (Mueller et al., 2013; Liu et al., 2014). JAM-A activity promotes migration of PMNs into the alveolar space, also relevant to increased oxidative stress (Lakshmi et al., 2012). Vasodilator-stimulated phosphoprotein (VASP) is colocalized with ZO-1, occludin, and JAM-1 and may favor PMN transmigration (Comerford et al., 2002). Antihuman JAM mAbs and high-titer polyclonal mouse antiserum generated against recombinant JAM seem to show no functional effect on TEpM, TEpM in the reverse direction, and PMN transmigration across human microvascular endothelial cell monolayers (Liu et al., 2000). Khandoga et al. first identified JAM-A as an endothelial receptor of neutrophil transmigration (Khandoga et al., 2005). PMN infiltration elevated, and the recruitment of leukocytes enhanced in the colonic mucosa of *Jam-A* ^−/−^ mice (Laukoetter et al., 2007). In uterine, mucosal epithelial cells stimulated with LPS and palmatine downregulated expression of JAM1 and could therefore facilitate the TEpM of leukocytes residing in the endometrium, such as neutrophils and macrophages (Hui et al., 2020). Distinguishing from JAM-A, PECAM-1 mediates migration through the endothelial-cell basement membrane when leukocyte PECAM-1 and endothelial-cell PECAM-1 to the same extent regulated *via* upregulated integrins α6β1 (Dangerfield et al., 2002; Woodfin et al., 2007). The Parkos team discovered that PMN migration into the colonic lumen was reduced in *Jam-A* ^*−/−*^ mice and *Villin-Cre*; *Jam-A* ^*fl/fl*^ mice, along with reduced peritoneal PMN migration in *Villin-Cre*; and *Jam-a* ^*fl/fl*^ mice (Flemming et al., 2018; Luissint et al., 2019; Boerner et al., 2021).

JAM-C mAbs and JAM-C/Fc chimeras significantly inhibited neutrophil transmigration, connected with the specific binding of JAM-C to the leukocyte *β2*-integrin Mac-1 (*α*M*β2*, CD11b/CD18) (Zen et al., 2004). Furthermore, LFA-1/Mac-1-JAM-C bonds can accelerate PMN crawling under high shear stress (Li G. C et al., 2018). Orlova et al. discovered that soluble JAM-C decreased leukocytes TEM and endothelial permeability by modulating VE-cadherin-mediated cell–cell contacts, working together with inhibition of Mac-1 (Chavakis et al., 2004; Orlova et al., 2006). However, Sircar et al. reported that JAM-C had a minimal role in neutrophil transmigration under shear flow conditions *in vitro* (Sircar et al., 2007). PMN TEM plays a considerable role in various inflammatory diseases. Transgenic mice overexpressing JAM-C under the control of the endothelial-specific promotor Tie2 showed increased leukocyte adhesion and transmigration to inflammatory sites (Aurrand-Lions et al., 2005). Soluble mouse JAM-C reduced neutrophil emigration in the mouse with acute thioglycollate-induced peritonitis and selectively reduced neutrophil infiltration into inflamed joints (Chavakis et al., 2004; Palmer et al., 2007). Cold-inducible RNA-binding protein (CIRP) induces neutrophil reverse transendothelial migration (rTEM) in sepsis by increasing neutrophil elastase (NE) and decreasing JAM-C (Jin et al., 2019). Blockade of JAM-C reduced the aged pro-inflammatory neutrophils in sepsis-induced acute lung injury (ALI), and JAM-C downregulation may contribute to acute pancreatitis (AP)-associated ALI *via* promoting neutrophil rTEM (Wu et al., 2016; Hirano et al., 2018). NE local proteolytically cleaved EC JAM-C *via* Mac-1 and therefore drove the lipid chemoattractant leukotriene B4 (LTB4) causing loss of venular JAM-C and promoting neutrophil reverse transendothelial cell migration (rTEM) *in vivo* (Colom et al., 2015). JAM-C was regarded as a negative regulator of rTEM under conditions of ischemia–reperfusion (I-R) and cisplatin-induced acute kidney injury (AKI) (Woodfin et al., 2011; Cho et al., 2017; Kim et al., 2017).

Zinc metalloproteases cleaved JAML from the neutrophil surface during PMN TEpM, and fusion proteins containing JAML and CAR extracellular domains and antibodies against JAML and CAR inhibited TEpM (Zen et al., 2005; Weber et al., 2014). Zen et al. proposed a revised model of PMN TEpM: sequential Mac-1-mediated binding to JAM-C at desmosomes when PMN cross the TJ, followed by JAML binding to CAR (Zen et al., 2005).

### Monocytes and Macrophages

A JAM-A mAb, BV11, was able to block human monocyte migration across bEND-3 cell monolayers *in vitro* and in the skin inflammatory and meningitis model *in vivo* (Martìn-Padura et al., 1998; Del Maschio et al., 1999; Williams et al., 1999). Nevertheless, both a monoclonal antibody and polyclonal rabbit IgG to JAM-A decreased slightly in monocyte transmigration (Liu et al., 2000; Schenkel et al., 2004). Activation of lung vascular endothelial ADAM17 and ADAM10 markedly promotes ectodomain shedding of JAM-A to enhance total leukocyte and neutrophil recruitment by LFA-1- and JAM-A-dependent mechanisms (Koenen et al., 2009; Dreymueller et al., 2012). Meanwhile, matrix metalloproteinase (MMP) contributes to the HIV-induced decreased expression of JAM-A and occludin, associated with elevated TEM of HIV-infected monocytes across an *in vitro* model of the blood–brain barrier (BBB) (Huang et al., 2009). Except for those functions of metalloproteinase in the previous content, Williams et al. found that CD14^+^CD16^+^ monocytes selectively transmigrated across the BBB model due to increased JAM-A and ALCAM expression in HIV-infected individuals (Williams et al., 2013; Williams et al., 2015). HIV+ CD14^+^CD16^+^ ART-treated monocytes (mature monocytes infected with HIV and treated with ART) preferentially transmigrate across the BBB to CCL2, which was reduced and/or blocked by blocking antibodies against junctional proteins JAM-A significantly (León-Rivera et al., 2021). In addition, buprenorphine limits the chemokine (C–C motif) ligand 2 (CCL2)-mediated monocyte transmigration into the central nervous system (CNS), through decreasing the phosphorylation of the junctional protein JAM-A increase (Carvallo et al., 2015). Also, this process probably links to CCL2-induced JAM-A redistribution *via* RhoA and Rho kinase (Stamatovic et al., 2012). JAM-1 is necessary for cellular interactions during β2-integrin-dependent leukocyte adhesion and transmigration on the inflammatory endothelium (Chavakis et al., 2003). It was found earlier that monocyte arrest and transmigration attenuated on activated *Jam-A* ^*−/−*^ *ApoE* ^*−/−*^ versus *Jam-A* ^*+/+*^ *ApoE* ^*−/−*^ endothelial cells under flow conditions *in vitro* (Zernecke et al., 2006). Then, Schmitt et al. found an endothelium-specific deficiency in JAM-A reduced mononuclear cell recruitment into the arterial wall, whereas somatic deficiency in JAM-A revealed no significant effects, accompanying endothelial JAM-A increased by oxidized low-density lipoprotein (oxLDL) but repressed by microRNA (miR)-145 (Schmitt et al., 2014a; Schmitt et al., 2014b; Liu et al., 2015). Tantalizingly, recruitment of platelets and monocytes to the inflamed endothelium increased in the blood of platelet-specific (tr)JAM-A-deficiency *ApoE* ^*−/−*^ mice, benefitted from αIIbβ3 signaling and the GPIbα–αMβ2 axis (Karshovska et al., 2015; Zhao et al., 2017). Ginkgolide B decreased the expression of JAM-A and reduced monocyte transmigration in oxLDL-treated HUVECs, linked to the attenuation of Akt phosphorylation (Liu et al., 2015). Additionally, p-cresol-impaired leukocyte TEM is potentially attributable to reduced membrane expression of JAM-A (Faure et al., 2006). Deep hypothermia and post-hypothermic rewarming regulate leukocyte–endothelial interaction and TEM, associated with JAM-A surface expression (Bogert et al., 2016; Bogert et al., 2020).

Liver irradiation does not lead to recruitment of leukocytes into the parenchyma, possibly related to the radiation-induced increase of JAM-1gene expression in rat livers *in vivo* and in hepatocytes *in vitro* (Moriconi et al., 2009). By the way, soluble JAM-C increased motility in hepatic stellate cells (Hintermann et al., 2016). Morphine, methamphetamine (Meth), and morphine- and tat-treatment significantly increased JAM-2 expression, while gp120 alone and in combination with Meth significantly decreased JAM-2 expression (Mahajan et al., 2008a; Mahajan et al., 2008b). All those treatments enhanced the TEM of immunocompetent cells across the BBB. Furthermore, JAM-C Fc chimera inhibited macrophage transmigration across hRPE (Hou et al., 2012). Blocking JAM-C function or JAM-B/-C interaction increased monocyte reverse transmigration in the peritonitis model (Bradfield et al., 2007). Consistently, monoclonal antibodies directed against JAM-C significantly blocked the influx of leukocytes in which cerulein-induced acute pancreatitis was assessed (Vonlaufen et al., 2006). Leukocyte transmigration was suppressed in *Jam-C* ^*−/−*^ mice and enhanced in mice overexpressing JAM-C in their ECs (Scheiermann et al., 2009). Similar to antibody blockade, overexpression or gene silencing of JAM-C in human endothelium exposed to flow elevated rates of monocyte reverse-transendothelial migration under inflammatory conditions *in vitro* (Bradfield et al., 2016). Neutralizing antibodies against JAM-C enhanced U937 cell migration through the rheumatoid arthritis (RA) synovial tissue fibroblast monolayer (Rabquer et al., 2008). JAM-C is upregulated by oxLDL and may thereby mediate both leukocyte adhesion and leukocyte TEM (Keiper et al., 2005b). Consequently, blocking JAM-C can assist the emigration of atherogenic monocytes/macrophages in plaques. In addition, exosomal miR-146a-5p that transported into endothelial cells reduced monocyte TEM by binding to the 3’untranslated region (3′UTR) of JAM-C (Hu et al., 2020). Monocytic JAML regulated TEM and TEpM of monocyte-derived THP-1 cells probably *via* binding to CAR, and this interaction is controlled by phosphorylation of CAR (Guo et al., 2009; Morton et al., 2016). During relapsing–remitting MS (RRMS), JAML has homophilic interaction with the BBB endothelium and heterophilical binding to CAR on the choroidal epithelium forming the blood–CSF barrier, which encourages monocyte and CD8 T-cell migration into the CNS (Alvarez et al., 2015).

### Dendritic Cell

JAM-A deficiency selectively increased DCs random motility and transmigration across lymphatic endothelial cells *in vitro* and enhanced DC migration to lymph nodes *in vivo* (Cera et al., 2004). H33, a monoclonal antibody against mouse JAM-C, improved the migration of DCs to sites of infection and in draining lymph nodes (Ballet et al., 2014).

### Lymphocytes

Under inflammation of the vascular wall, JAM-A mediated lymphocyte recruitment to the endothelium and subsequent TEM, through redistribution of JAM-A receptors toward endothelial junctions (Jaczewska et al., 2014). JAM-1 knockdown of EC inhibited chemokine-dependent TEM of effector memory (EM) CD4^+^ T cells (Manes and Pober 2011). Under flow conditions, stromal cell-derived factor (SDF)-1alpha triggered transendothelial chemotaxis of activated T cells and arrest on cytokine-costimulated endothelium, which could be inhibited by soluble JAM-A.Fc (sJAM-A.Fc) (Ostermann et al., 2005). Blocking EZH2 or JAM-A reduced T-cell adhesion, migration, and extravasation, and EZH2 was involved in leukocyte adhesion and migration *via* upregulating JAM-A (Tsou et al., 2018). VE-JAM (JAM-B) was prominently expressed in HEV and distributed at interendothelial boundaries (Palmeri et al., 2000). The Engelhardt team discovered JAM-B, implicated in CD8 T-cell migration to the CNS, as one ligand for α4β1-integrin, but JAM-B deficiency does not affect T-cell transmigration across the BBB *in vitro* (Martin-Blondel et al., 2015; Tietz et al., 2018). JAM-2 promotes lymphocyte TEM (Johnson-Léger et al., 2002). Recombinant JAM-C binds to Mac-1 on BM-DCs (Zimmerli et al., 2009). Although JAM-C was upregulated in activated human T lymphocytes (Immenschuh et al., 2009), JAM-C-deficiency did not affect DC homing, T-cell activation, and DC migration to lymph nodes (Zimmerli et al., 2009). JAM-C potentially works in the final steps of trafficking and transmigration of antigen-specific autoaggressive T-cells to the islets of Langerhans (Christen et al., 2013). Anti-JAM-C antibodies could reduce migration of normal and malignant JAM-C-expressing B cells to bone marrow, lymph nodes, and spleen, probably associated with blockage of adhesion to their ligand JAM-B (Doñate et al., 2013). Not only that, endothelial-cell-selective adhesion molecule (ESAM)-1, an endothelial TJ protein linked with the JAMs, was predicted to attend cell migrations through the sinus-lining cell layer (Pfeiffer et al., 2008).

**FIGURE 1:**
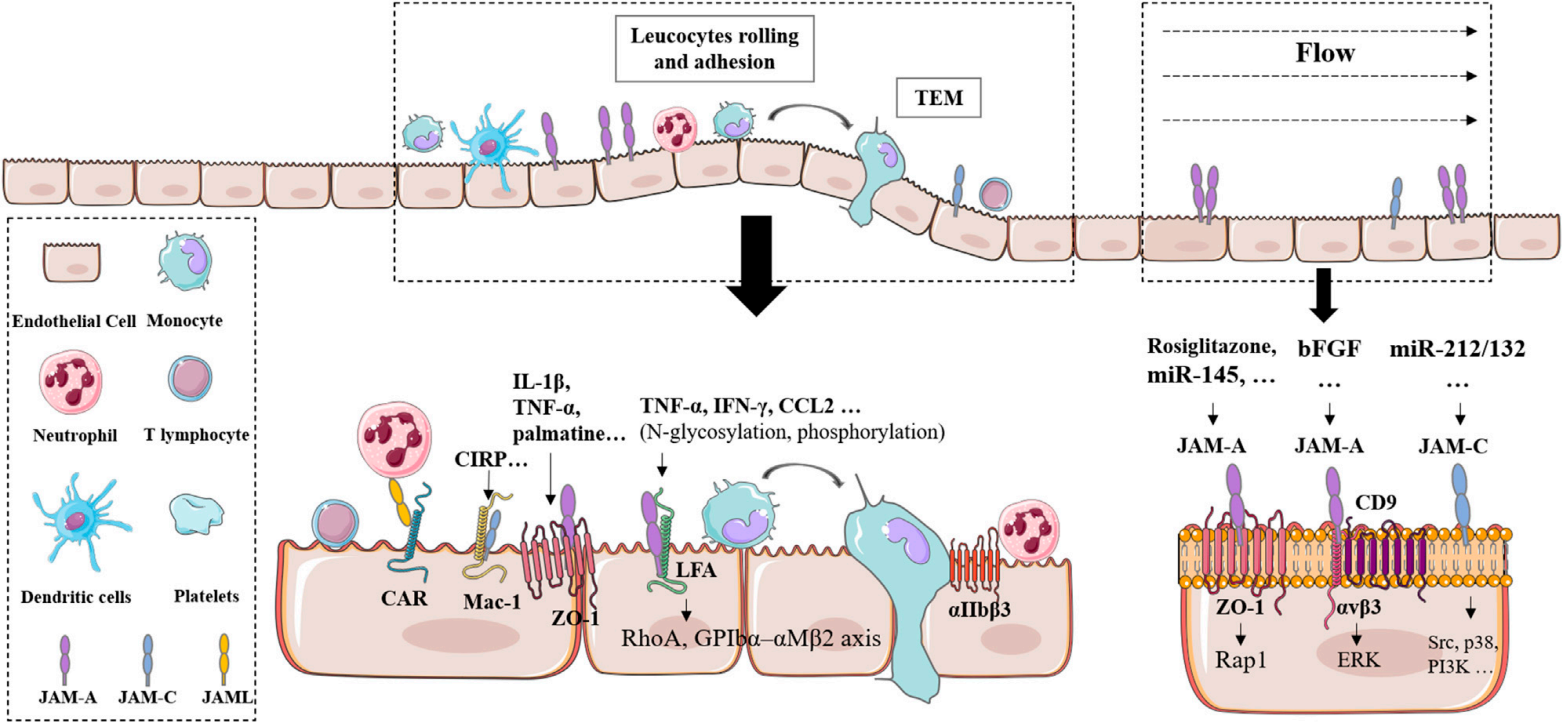
| Roles of JAMs on endothelial cell migration and leucocytes TEM.

Taken together, JAMs have been recognized as important players controlling leukocyte transendothelial migration. According to the notable functions of TEM in various physiological and pathological situations, JAMs play complicated and variable roles (Figure 1).

Representative interaction and signaling of JAMs. Abbreviation is attached at the end.

## Cancer

A couple of reviews summarized the function of JAMs in cancer in recent years, and this review focused on their roles in cancer invasion and metastasis.

Naik et al. discovered JAM-A expression was downregulated as breast cancer disease progresses and thereupon enhanced cancer cell migration (Naik et al., 2008; Naik and Naik 2008). McSherry et al. observed that knockdown or functional antagonism of JAM-A drove breast cancer cell migration *via* activation of Rap1 GTPase and β1-integrin (McSherry et al., 2009; McSherry et al., 2011). In the murine 4T1 breast cancer model, administration of Tβ4 and TGF-β1 decreased the sJAM-A levels in murine blood, and a peptide derived from the sequence of the F11R/JAM-A protein, peptide 4D (P4D), blocked the TEM of breast cancer cells in the presence of TNF/IFN and Tβ4 (Bednarek et al., 2020). In human breast cancer cell lines, JAM-A blocked the pro-migratory function of CD146 (Imbert et al., 2012) and connected a unique antihuman CD81 antibody (5A6), which effectively halts tumor cell invasion and migration (Vences-Catalán et al., 2021). MicroRNA-495 stimulated breast cancer cell migration by targeting JAM-A (Cao et al., 2014). In the Rip1Tag2 tumor model, *Jam-A* ^−/−^ DCs had a higher rate of DC migration through the endothelium into the tumor than *Jam-A* ^*+/+*^ DCs (Murakami et al., 2010). High JAM-A expression induces EMT of nasopharyngeal carcinoma (NPC) cells *in vitro* and *in vivo via* the PI3K/Akt pathway, and lncRNA P73 antisense RNA 1 T (TP73-AS1) could upregulate JAM-A expression (Tian et al., 2015; Dai et al., 2021). Histone deacetylases (HDACs) inhibitors downregulated p63-mediated JAM-A expression, suppressing the proliferation, migration, and invasiveness of human head and neck squamous cell carcinoma (HNSCC) (Kakiuchi et al., 2021). Aberrant expression of JAM-A regulated PVR/CD155 to exacerbate malignancy of uterine cervical adenocarcinoma (Murakami et al., 2021). JAM-A restoration suppressed anaplastic thyroid carcinoma (ATC) cell motility and TEM, related to the level of phosphorylation of p53 and GSK3 α/β proteins (Orlandella et al., 2019). In colorectal cancer (CRC), MIR21 upregulation caused JAM-A downregulation and then activated ERK, AKT, and ROCK pathways in promoting invasiveness and metastasis (Lampis et al., 2021). Furthermore, JAM-A expression declined in renal cancer (Gutwein et al., 2009), gastric cancer (Huang et al., 2014), and multiple myeloma (MM) (Solimando et al., 2018) and impaired these cancer cells migration and invasion. Incidentally, N-glycosylation controlled JAM-A’s effects on the migration of MDA-MB-231 cells (Scott et al., 2015).

Qi et al. reported quantum dots (QDs) or cell-penetrating magnetic nanoparticles-mediated JAM-2 knockdown facilitated inhibition of glioma cell migration, and JAM-2 gene targeted the Notch pathway and regulated cytoskeleton remodeling and migration associated protein gene expression (Qi et al., 2013; Qi et al., 2014). Over-expression of JAM-2 in RKO cells resulted in decreasing growth, migration, adhesion, and invasion by regulating the transcription of MMP-9. Negatively binding of miR-374b and JAM-2 inhibits cervical cancer (CC) cell proliferation, migration, and invasion (Li X et al., 2018). JAM-B secreted by cancer cells could promote progression and invasion in pancreatic cancer (PanCa) by upregulating the c-Src signal and related downstream proteins (Zhang et al., 2020).

JAM-C dephosphorylation at serine 281 increased KLN 205 cell adhesion and migration by activating β3 integrins and deactivating β1 integrins (Mandicourt et al., 2007). In Lewis lung carcinoma cells (LLC1s), treatment with a monoclonal antibody directed against JAM-C reduced the infiltration of macrophages into tumors (Lamagna et al., 2005). Palmitoylation of JAM-C supported the movement to TJs and inhibited A549 lung cancer cells migration (Aramsangtienchai et al., 2017). JAM3 endorsed CRC cell viability, colony formation, and migration (Zhou et al., 2019). Some researchers from Geneva University Hospital recounted JAM-C function in several cancers. Anti-JAM-C antibodies reduced migration of normal and malignant JAM-C-expressing B cells to bone marrow, lymph nodes, and spleen by blocking adhesion of JAM-C-expressing B cells to ligand JAM-B (Doñate et al., 2013) and impaired lymphoma B-cell homing to supportive lymphoid microenvironments by driving the MAPK signaling pathway (Doñate et al., 2016). JAM-C/B combination escalated glioma growth and invasion *in vivo*, linked to activated c-Src proto-oncogene (Tenan et al., 2010). The dimerization sites E66-K68 of JAM-C affected mouse lung squamous carcinoma KLN 205 cells migration (Garrido-Urbani et al., 2018). Moreover, circKIF4A promoted cell proliferation and migration in ovarian cancer by sponging miR-127 and upregulating JAM3 expression (Sheng et al., 2020). B16 melanoma cell metastasis to the lung was proved to decrease in *Jam-C* ^*−/−*^ mice and endothelial-specific JAM-C-deficient mice, and treatment with soluble JAM-C created a similar decrease (Langer et al., 2011). JAM-C also promoted HT1080 human fibrosarcoma metastasis (Fuse et al., 2007). However, JAM-3 was proved to suppress migration and promote apoptosis of renal carcinoma cells (Li G. C et al., 2018).

JAML promoted gastric cancer (GC) cell migration and proliferation partially *via* p38 signaling (Fang et al., 2021). In DC-based cancer immunotherapy, the interaction of JAML and CAR acted a crucial role in the TEM of mouse bone marrow-derived DCs (BMDCs) and human monocyte-derived DCs (MoDCs) (Roh et al., 2018).

In fact, numerous articles depicted the significant function of JAMs in cancer notwithstanding, but there are still many questions. For instance, JAM-A diminished the SLM8 cells line TEM, but JAM-C enhances the A375 cell line TEM conversely (Ghislin et al., 2011). Hence, figuring out how JAMs function in different cancer requires further investigation. The roles of JAMs in cancers have been concluded in Table 3.

**TABLE 3:** JAMs in cancers.

Protein	Cancer	Final effect
JAM-A	Breast cancer, anaplastic thyroid carcinoma, and colorectal cancer	Inhibits invasion and migration
		Naik et al. (2008)
		Naik and Naik (2008)
		McSherry et al. (2009)
		McSherry et al. (2011)
		Bednarek et al. (2020)
		Imbert et al. (2012)
		Vences-Catalán et al. (2021)
		Cao et al. (2014)
		Orlandella et al. (2019)
		Lampis et al. (2021)
	Nasopharyngeal carcinoma, head and neck squamous cell carcinoma, uterine cervical adenocarcinoma, renal cancer, gastric cancer, and multiple myeloma	Induces proliferation, migration, and invasiveness
		Tian et al. (2015)
		Dai et al. (2021)
		Kakiuchi et al. (2021)
		Murakami et al. (2021)
		Gutwein et al. (2009)
		Huang et al. (2014)
		Solimando et al. (2018)
JAM-B	Glioma and pancreatic cancer	Promotes progression and invasion
		Qi et al. (2013)
		Qi et al. (2014)
		Zhang et al. (2020)
JAM-C	Ovarian cancer, melanoma, and fibrosarcoma metastasis	Promotes cell proliferation and migration
		Sheng et al. (2020)
		Langer et al. (2011)
		Fuse et al. (2007)
	Renal carcinoma	Suppresses migration
		Li N et al. (2018)
JAML	Gastric cancer	Promotes migration and proliferation
		Fang et al. (2021)

## Conclusion and Prospective

Multiple research studies implicated the principal suppressive effect of JAMs in cell migration with tight junctions. Related inhibitory action was presumably due to the conjunction with the ZO family of scaffolding proteins ZO-1 (Garcia et al., 2018), claudin, afadin (Keiper et al., 2005a; Hartmann et al., 2020; Ebnet 2017), Par6 (Gliki et al., 2004), Par-3 (Fujita et al., 2007), and several integrins (Parise 2003; Naik et al., 2003; Cooke et al., 2006; Peddibhotla et al., 2013; Zen et al., 2004; Karshovska et al., 2015; Zhao et al., 2017; Martin-Blondel et al., 2015; Tietz et al., 2018) (Figure 1). Direct downstream proteins included one integrin ligand such collagen I, collagen IV, and fibronectin (Ebnet 2017).

Another point that deserved discussion should be shear flow, which is important for the migration of endothelial cells as well as leukocytes. Under flow conditions, JAM-A deficiency increased protrusion extension in the direction of flow and enhanced downstream cellular displacement by cooperating with microtubule-stabilizing pathways in ECs (Huang et al., 2006). Whereas, JAM-A deficiency and soluble JAM-A.Fc attenuated monocyte and activated T cells arrest and transmigration (Ostermann et al., 2005; Zernecke et al., 2006). Under shear flow conditions, the antibody against JAM-C scarcely influenced neutrophil transmigration yet elevated rates of monocyte reverse-transendothelial migration *in vitro* (Sircar et al., 2007; Bradfield et al., 2016).

**FIGURE 2:**
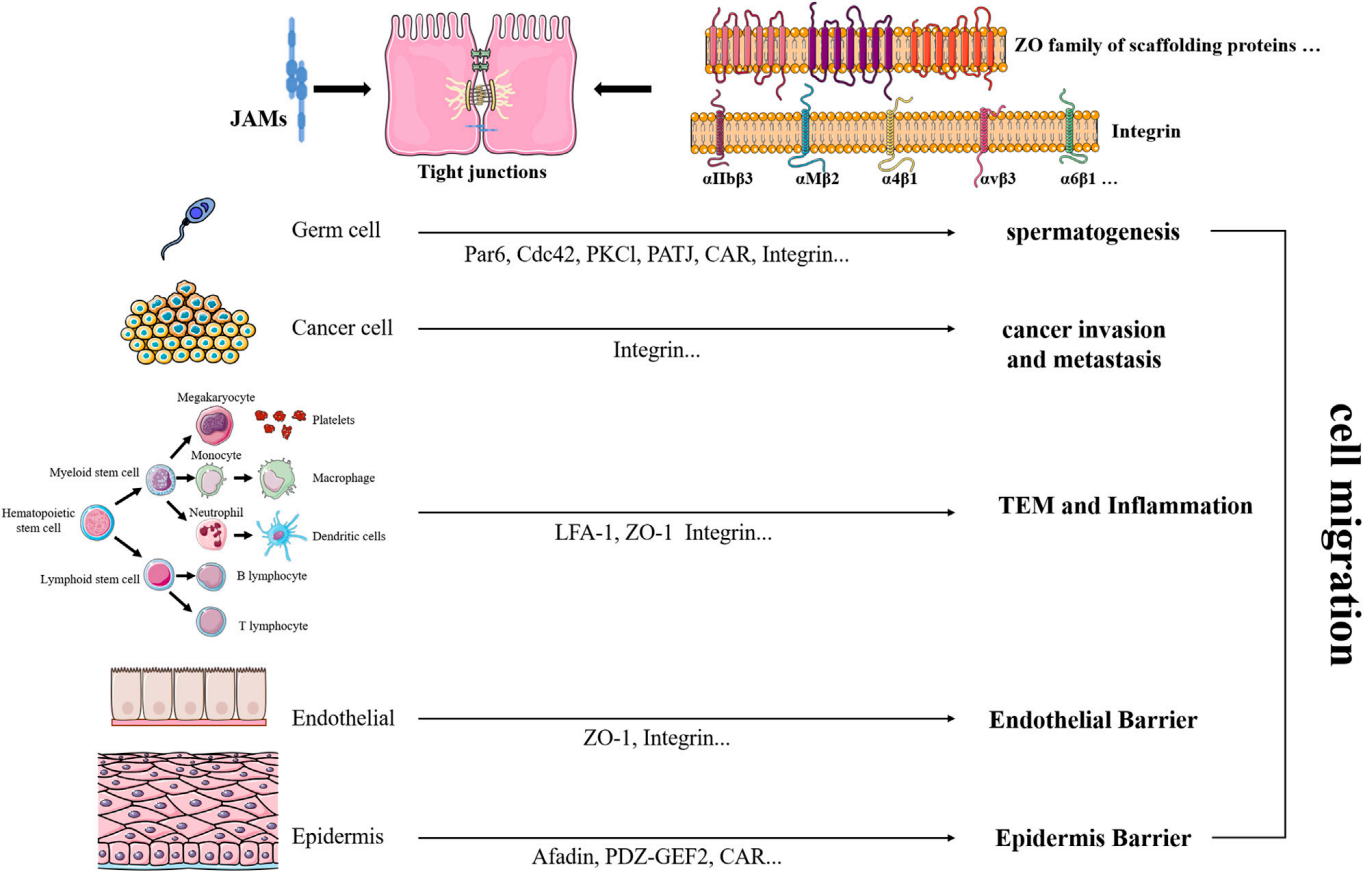
| Regulatory roles of JAMs on cell migration.

In addition to the cells described previously, there was a rare report in the central nervous system. For example, Pard3A-dependent JAM-C adhesion promotes germinal zone (GZ) exit OF neuronal cells (Famulski et al., 2010). Nevertheless, JAMs exhibited distinct even opposite functions in different physiological and pathological activities (Ghislin et al., 2011). Since JAMs exhibit unclear roles in one specific cell type, it is more difficult to define their roles under one physiological state or pathological condition. At present, soluble JAMs, the antibody against JAMs, and Jam-deficient mice were developed and were favorable to follow-up studies and potential application. These results remind us that further and detailed examination is necessary for explaining specific influences. Figure 2.

## Abbreviations and Glossary

AJs: adherens junctions
AKI: acute kidney injury
ALI: neutrophil elastase, acute lung injury
AP: acute pancreatitis
ATC: anaplastic thyroid carcinoma
BBB: blood–brain barrier
BG: bioactive glass
BMDCs: bone marrow-derived DCs
BMEC: brain microvascular endothelial cells
BTB: blood–testis barrier
C5aR: complement factor 5
CBL: Casitas B-lineage lymphoma
CC: cervical cancer
CCL2: (C–C motif) ligand 2
CIRP: cold-inducible RNA-binding protein
CIS: carcinoma *in situ*
CNP: C-type natriuretic peptide
CNS: central nervous system
CPA: cyproterone acetate
CPCs: cardiac progenitor cells
CRC: colorectal cancer
ECs: endothelial cells
e-EPCs: embryonic-endothelial progenitor cells
EM: effector memory
ES: ectoplasmic specialization
ESAM: endothelial-cell-selective adhesion molecule
GC: gastric cancer
GCs: germ cells
GZ: germinal zone
HDACs: histone deacetylases
HF: hair follicle
HMVEC: human microvascular endothelial cell
HNSCC: head and neck squamous cell carcinoma
HRCECs: human retinal capillary endothelial cells
I-R: ischemia–reperfusion
JAMs: junctional adhesion molecules
JAM-A(ov) MSCs: JAM-A overexpression MSCs
JAM-Ap: JAM-A antagonist peptide
LFA-1: leukocyte function-associated antigen-1
LLC1: Lewis lung carcinoma cells
LTB4: leukotriene B4
MCCs: multiciliated cells
Meth: methamphetamine
MM: multiple myeloma
MMP: matrix metalloproteinase
MoDCs: monocyte-derived DCs
NAC: N-acetyl-l-cysteine
NPC: nasopharyngeal carcinoma
NRSF: neuron-restrictive silencer factor
oxLDL: oxidized low-density lipoprotein
PanCa: pancreatic cancer
P4D: peptide 4D
P-gp, MDR1: P-glycoprotein
pSp1: proximal Sp1
QDs: quantum dots
RA: rheumatoid arthritis
RGDS: Arg-Gly-Asp-Ser
RPE: retinal pigment epithelial
RRMS: relapsing–remitting MS
rTEM: reverse transendothelial migration
SCs: Sertoli cells
SDF: stromal cell-derived factor
sJAM-A.Fc: soluble JAM-A.Fc
Smad: mothers against decapentaplegic
SPARC: secreted protein acidic and rich in cysteine
TEM: transendothelial migration
TEpM: transepithelial migration
TGIF: TG-interacting factor
Tiam1: T-cell lymphoma invasion and metastasis 1
TJs: tight junctions
TP73-AS1: lncRNA P73 antisense RNA 1 T
3′UTR: 3’untranslated region
VASP: vasodilator-stimulated phosphoprotein
